# Systematic comparison of approaches to analyze clustered competing risks data

**DOI:** 10.1186/s12874-023-01908-6

**Published:** 2023-04-10

**Authors:** Sabrina Schmitt, Anika Buchholz, Ann-Kathrin Ozga

**Affiliations:** 1 Koblenz University of Applied Science, RheinAhrCampus Remagen, Joseph-Rovan-Allee 2, 53424 Remagen, Germany; 2 Institute of Medical Biometry and Epidemiology, University Medical Center Hamburg-Eppendorf, Martinistraße 52, 20246 Hamburg, Germany

**Keywords:** Competing events, Time-to-event, Cluster

## Abstract

**Background:**

In many clinical trials the study interest lies in the comparison of a treatment to a control group regarding a time to event endpoint like time to myocardial infarction, time to relapse, or time to a specific cause of death. Thereby, an event can occur before the primary event of interest that alters the risk for or prohibits observing the latter, i.e. a competing event. Furthermore, multi-center studies are often conducted. Hence, a cluster structure might be observed. However, commonly only the aspect of competing events or the aspect of the cluster structure is modelled within primary analysis, although both are given within the study design. Methods to adequately analyze data in such a design were recently described but were not systematically compared yet.

**Methods:**

Within this work we provide a systematic comparison of four approaches for the analysis of competing events where a cluster structure is present based on a real life data set and a simulation study. The considered methods are the commonly applied cause-specific Cox proportional hazards model with a frailty, the Fine and Gray model for considering competing risks, and extensions of the latter model by Katsahian et al. and Zhou et al.

**Results:**

Based on our simulation results, the model by Katsahian et al. showed the best performance in bias, square root of mean squared error, and power in nearly all scenarios. In contrast to the other three models this approach allows both unbiased effect estimation and prognosis.

**Conclusion:**

The provided comparison and simulations help to guide applied researchers to choose an adequate method for the analysis of competing events where a cluster structure is present. Based on our simulation results the approach by Katsahian et al. can be recommended.

**Supplementary Information:**

The online version contains supplementary material available at 10.1186/s12874-023-01908-6.

## Background

The aim of many clinical trials is the comparison of two treatment groups regarding the occurrence of one specific event among many competing events. For example, a specific cause of death is of primary interest but another absorbing event (like another cause of death) occurs before the primary event of interest and hence the latter cannot be observed. Furthermore, clinical trials are often conducted at multiple treatment centers, which leads to a cluster structure. Commonly, the Cox proportional hazards model [1] is applied to investigate the time until the first event takes place, i.e. cause-specific analysis is applied. In this model, competing events are censored, while the treatment effect on the probability of occurrence of the event of interest is estimated [2]. Therefore, there is only one possible event during the estimation process. However, this can lead to an invalid analysis of the cumulative probabilities. Another frequently used approach is the Fine and Gray model [3] where individuals experiencing a competing event are not censored but remain in the risk set for the primary event of interest. The treatment effect is now estimated using a subdistribution hazard. Existing cluster structures are still mostly ignored in this evaluation of clinical trials with competing risks. This is considered problematic because it is assumed that existing cluster structures lead to a dependency of failure times within clusters [4, 5]. Ignoring these correlations might result in underestimation of variance of the group-specific regression parameters. Therefore, to address this problem, new approaches which analyze event times in competing risks settings that also take cluster structures into account, are needed. Usually competing risks are defined by the following properties:

- There are multiple events possible, but only one can occur at a time.
- In clinical trials, one event is always of primary interest.
- In general, researchers are interested in the first time the event occurs.

The aim of this work is therefore to compare the performance of different models used for analyzing survival data in settings with competing risks regarding their accuracy in the presence of cluster structures. In this paper, we compare two newer approaches by Katsahian et al. [4, 5] and Zhou et al. [6] which explicitly address this topic and contrast them to the commonly used Fine and Gray model [3] which addresses competing risks but not the cluster structure as well as the cause-specific Cox proportional hazards based model with a frailty term [7–9] which incorporates the cluster structure but ignores the presence of competing risks. However, in contrast to Katsahian et al. and Zhou et al. we are mainly interested in the effect estimation and not prognosis, i.e. we consider bias, square root of mean squared error, and empirical power for the estimated treatment effect and do not consider the performance of the models regarding prognosis. This is due to our focus on clinical trials where the main aim is to estimate a treatment effect. Hence, it is evaluated whether the models by Katsahian et al. and Zhou et al. can also be used for unbiased effect estimation and not only for prognosis. If both, effect estimation and prognosis, could be possible with one model this would be optimal.

The approach by Katsahian et al. uses an alternative estimation method, which has already been used in the context of standard survival analysis and has now been extended for competing risks settings. In addition, it uses a specific weighting technique by which individuals who have already experienced an event from a competing cause are weighted in the model. Individuals are considered to be at risk until they experience either the event of interest or censoring. The main interest of the approach by Zhou et al. is to assess marginal effects of covariates on the cumulative incidence function for the occurrence of an event of interest conditional on the covariates. The strength of this model is, that it takes into account both dependencies of failure times and censoring times.

The performance of the models is examined and compared using a Monte Carlo simulation study considering different relevant clinical settings. As mentioned above, as performance measures bias, square root of mean squared error, and empirical power for the effect estimation are considered.

Hence, we provide a neutral comparison as proposed by [10, 11]. Thereby, we highlight the properties of commonly used methods in the context of clustered competing risk data, as well as methods that have been proposed but are not used in clinical practice to analyze such data. The Cox proportional hazards model with frailty was chosen because it is the most frequently applied method in clinical trials where a time to event endpoint is of interest and a cluster structure is present due to different clinical centers involved. Although the competing risks structure is not considered in this method, it has been recommended for the primary analysis of randomized controlled trials [12]. The Fine and Gray model is of interest, since it is commonly used for the analysis of survival data where competing events are present. We wanted to investigate to which extend this method, although theoretically not fully appropriate for the setting at hand, differs from the ’correct’ methods or whether it might yield acceptable results in practice. The two ’newer’ methods were chosen because they were proposed for the analysis of clustered competing risk data and model a hazard ratio. Other methods that were described in the literature were additive models [13] or models with copulas [14]. The additive model does not result in a hazard ratio and is thus not comparable to the other methods [13]. Furthermore, additive models are not as common as multiplicative models in survival analyses. The copula approach produces an estimate which describes the degree of acceleration which was not our main interest [14].

This paper is structured as follows: We will start by the separate presentation of the statistical models. Afterwards we will present and compare the obtained simulation results of each model. In the end we discuss the methods and results and finish the article with a conclusion.

## Methods

We consider a two-arm multi-center clinical study with an intervention *I* and a control *C*, where the primary endpoint (*PE*) is a time to event endpoint. Further, we assume that a competing event (*CE*) might be observed. The competing event might occur before the primary event of interest. Moreover, adminstrative censoring is assumed. The individuals $$i=1,...,n$$ are randomized in a 1 : 1 allocation to the two groups within each center ($$k=1,...,K)$$, where K is the total number of centers (i.e. clusters). We consider a two-sided test problem, i.e. test for difference with

Null-hypothesis:

1 $$\begin{aligned} H_0: \beta _{PE} = 0 \end{aligned}$$

and

Alternative hypothesis:

2 $$\begin{aligned} H_1: \beta _{PE} \ne 0. \end{aligned}$$

Thereby, $\beta _{PE}=log(HR_{PE})$ denotes the treatment effect for the primary endpoint of interest (HR = hazard ratio).

The following sections describe the methods evaluated in this work.

### Cox proportional hazards model with frailty

In the Cox proportional hazards model [1] the hazard for an individual $i=1,...,n$ within a two-group comparison is modelled as follows:

3 $$\begin{aligned} \lambda _{i}(t)=\lambda _{0}(t)exp(\beta _{PE} X_i) \end{aligned}$$

where $\lambda _{0}(t)$ refers to a common cause-specific baseline hazard, i.e. the instantaneous baseline risk for an individual to experience an event of one specific type (primary event of interest) at time *t* given no prior event. $X_i$ is the treatment indicator, i.e. $X_i$ = 1 if individual *i* belongs to the intervention group (*I*), and $X_i$ = 0 for the control group (*C*). $\beta _{PE}$ is the corresponding coefficient.

This model can be extended to model heterogeneity in the data due to e.g. clustered data like within a multi-center trial. This extension is given as follows [8, 9]:

4 $$\begin{aligned} \lambda _{i}(t)=\lambda _{0}(t)exp(\beta _{PE} X_{i}+u Z_i). \end{aligned}$$

This model is also called frailty model [7–9], where *u* are independent and identically distributed from some positive scale family with expected mean value of 0 and variance $\theta$. Within this work we assume a gamma distribution with $u\sim log(\Gamma (\frac{1}{\theta },\frac{1}{\theta }))$. $Z_i$ is a vector of indicator variables, i.e. $Z_i=1$ if an individual *i* belongs to the cluster of interest and otherwise $Z_i=0$.

In a competing risks setting this approach is used as cause-specific analysis where the competing event time point is assumed as a censored time point. Thereby, a cause-specific treatment effect can be given, i.e. it allows one to estimate the effect of the treatment on the rate of occurrence of the primary outcome of interest in those subjects who are currently event free. Within a frailty model the treatment effect is conditional on the frailty.

For effect estimation the maximum likelihood approach can be used.

The partial log-likelihood with gamma frailty is given as [8, 9]:

5 $$\begin{aligned} l^{Cox}= & {} \sum \limits _{i=1}^n \delta _i\Bigg ((\beta _{PE} X_{i}+ u Z_i)\nonumber \\{} & {} - log\left. \left( \sum \limits _{j\in R_i} exp(\beta _{PE} X_{j}+u Z_j )\right) \right) \nonumber \\{} & {} +\frac{1}{\theta }\sum \limits _{k=1}^K(u_k-exp(u_k)). \end{aligned}$$

Thereby, the risk set $R_i$ is defined at the time of failure $t_i$ for the *ith* individual as

6 $$\begin{aligned} \lbrace j: (t_j\ge t_i)\rbrace . \end{aligned}$$

$\delta _i$ indicates the event time point ($\delta _i=1$) or censoring time point ($\delta _i=0$). $u_k$ is the independent and identically distributed frailty for center *k*.

### Fine and Gray model

Fine and Gray [3] proposed a semi-parametric proportional hazards model for the subdistribution of a competing risk to assess the treatment effect on the marginal probability function. The hazard formulation for the primary event of interest is given as:

7 $$\begin{aligned} \lambda _{i,PE}(t)=\lambda _{0,PE}(t)exp(\beta _{PE} X_i). \end{aligned}$$

As before $X_i$ is the treatment indicator and $\beta _{PE}$ is the corresponding coefficient. The baseline subdistribution hazard for the primary event of interest is $\lambda _{0,PE}(t)$.

Within this model clustered data structure is not considered.

For effect estimation the partial log-likelihood can be used and is given as follows[3]:

8 $$\begin{aligned} l^{FG} =\sum \limits _{i=1}^n I(\epsilon _i=PE)\cdot \Bigg (\beta _{PE} X_i\\ \nonumber -log\left. \left( \sum \limits _{j\in R_i}exp(\beta _{PE} X_j)\right) \right) . \end{aligned}$$

Thereby, the risk set $R_i$ defined at the time of failure for the *ith* individual is given as

9 $$\begin{aligned} \lbrace j: (t_j\ge t_i)\cup (t_j\le t_i \cap \epsilon _j \ne PE)\rbrace \end{aligned}$$

with $\epsilon$ indicating the cause of failure and *t* the event times. Please note, that the risk sets, i.e. individuals at risk for an event, for this marginal approach and the cause-specific approach will usually not be the same.

### Approach by Katsahian et al.

In 2006 Katsahian et al. [5] introduced the random effects model for the subdistribution hazard as an extension of the classical Fine and Gray model. It offers the opportunity to consider clustered event data appropriately in the model. In contrast to the standard model, frailties and random center effects are now integrated into the subdistribution hazard, resulting in the following modification of the subdistribution hazard of the individual *i*:

10 $$\begin{aligned} \lambda _{i,PE}(t)=\lambda _{0,PE}(t)exp(\beta _{PE} X_i+u Z_i). \end{aligned}$$

As before $X_i$ is the treatment indicator, $\beta _{PE}$ the corresponding coefficient, and the baseline subdistribution hazard for the primary event of interest is $\lambda _{0,PE}(t)$. *u* is the frailty vector, where frailties are considered independent and normally distributed with mean 0 and variance $\theta$. $Z_i$ is a vector of indicator variables, i.e. $Z_i=1$ if individual *i* belongs to the cluster of interest and otherwise $Z_i=0$.

For estimation the following log-likelihood function is used [4]:

11 $$\begin{aligned} l^{Kat}= & {} \sum \limits _{i=1}^nI(\epsilon _i=PE)\Bigg ((\beta _{PE} X_i+uZ_i)\nonumber \\{} & {} -log\left. \left( \sum \limits _{j=1}^n w_j(t_i)exp(\beta _{PE} X_j+u Z_j)\right) \right) \nonumber \\{} & {} -\frac{1}{2}\left( K\cdot log(2\pi \theta )+\frac{u^{'}u}{\theta }\right) . \end{aligned}$$

Thereby

12 $$\begin{aligned} \omega _i(t)=I(t_i\ge t \cup \epsilon _i \ne PE)\frac{\hat{G}(t)}{\hat{G}(t_i\wedge t)}. \end{aligned}$$

In this formula $t_i$ describes the minimum of the observed failure time and the censoring time of the individual *i* and $t_i\wedge t$=$min(t_i,t)$. $\omega _i(t)$ describes the weighting of the individual *i* at time *t*, according to its censoring status, where $\hat{G}(\cdot )$ describes the Kaplan-Meier estimate of the survival function of the censoring times. This formula shows that individuals who did not experience an event before time *t* $(t\le t_i)$ are fully considered in the model ($\omega _1(t)$=1). In case of $t>t_i$ two situations must be distinguished:

- If individual *i* experienced an event ($\epsilon _i=PE$) or was censored prior to time *t*, the weight is zero ($\omega _i=0$).
- If individual *i* experienced a competing event prior to time *t*, the individual weighting is done using $\omega _i(t)=\frac{\hat{G}(t)}{\hat{G}(t_i\wedge t)}$. In summary, individuals who have experienced a competing event ($\epsilon _i = CE$) remain at risk until some kind of censoring occurs $C_i>t$ ($C_i$ denotes the censoring time of individual *i*).

### Approach by Zhou et al.

Zhou et al. [6] described a marginal proportional subdistribution hazards model which provides the ability to evaluate marginal effects of covariates on the cumulative incidence function. An existing correlation between individuals of the same cluster, due to unobserved factors, can be accounted for in settings of clustered competing risks. Within the marginal proportional subdistribution hazards model individuals within one cluster are considered as independent observations and the correlation between these individuals remains completely unspecified. Using the independence assumption, the Fine and Gray methodology can be used to estimate the cumulative incidence function and the effects of prognostic factors. However, the variance estimator allows not only the consideration of correlations between failure times but also the consideration of existing dependencies between the individual censoring times within a cluster. The variance estimation does not require any specification of the dependency between the individuals.

The main interest of the model is to assess the effect of the covariates on the marginal cumulative incidence function for the occurrence of event type *PE* conditional on the covariates. Thereby, the subdistribution hazard for *PE* is defined as:

13 $$\begin{aligned} \lambda _{i,PE}(t)=\lambda _{0,PE}(t)exp(\beta _{PE} X_{ik}). \end{aligned}$$

The baseline subdistribution hazard for the primary event of interest is $\lambda _{0,PE}(t)$. $X_{ik}$ is the indicator for an individual belonging to a specific treatment and cluster *k* with corresponding coefficient $\beta _{PE}$.

For effect estimation the log-likelihood is given as:

14 $$\begin{aligned} l^{Zhou}= & {} \sum \limits _{k=1}^K \sum \limits _{i=1}^{n_k} I(\epsilon _{ik} =PE)\Bigg (\beta _{PE} X_{ik}\nonumber \\{} & {} - log\left. \left( \sum \limits _{j\in R_{ik}} exp(\beta _{PE} X_{jk})\right) \right) . \end{aligned}$$

The number of individuals in a cluster is denoted by $n_k$. Risk set within a cluster *k* is defined via:

15 $$\begin{aligned} \lbrace j: (t_{jk}\ge t_{ik})\cup (t_{jk}\le t_{ik} \cap \epsilon _{jk} \ne PE)\rbrace . \end{aligned}$$

### Simulation study

To provide a systematic comparison of the methods described in the previous sections, we conducted a simulation study with the statistic software R (Version 4.0.2) [15]. We used the packages survival [16], coxme [17], and crrSC [18] for the analysis.

R uses the Mersenne twister [19] for generating random numbers.

We considered a competing risks setting with one primary event and one competing event. Within each proportional cause-specific hazards are assumed for the two groups, i.e. a constant treatment effect over time.

To gain first insights into the performance of the four methods we considered scenarios with independent competing events.

Since we assumed a multi-center trial, different cluster counts are considered, as well as stratified randomization (i.e. stratum = center). Within each center, i.e. a cluster, the observations might be more correlated than between clusters. To model this we assumed a cluster-specific additional parameter. To be more precise a gamma-distributed frailty is added to the common baseline hazard (see formula 4).

The event times are generated as described by Bender et al. [20] for the two event processes, i.e. each individual gets an event time for the primary event of interest and the competing event. Of those the earlier time is assigned to the individual. If both times are larger than the administrative censoring time point, this censoring time point is used. Within the approach by Bender et al. the center-specific frailty can be incorporated, see e.g. [21, 22].

**Table 1:** Simulation Scenarios

Scen.	$\lambda _{0,PE}(t)$	$\lambda _{0,CE}(t)$	$HR_{PE}$	$HR_{CE}$	$log(HR_{PE})$	$log(HR_{CE})$	$\theta$	cluster count
1*a*	$4\cdot t$	$4\cdot t$	0.5	0.7	$-0.69$	$-0.36$	0	5
1*b*								10
1*c*								25
2*a*	$4\cdot t$	$4\cdot t$	0.5	0.7	$-0.69$	$-0.36$	0.5	5
2*b*								10
2*c*								25
3*a*	$4\cdot t$	$4\cdot t$	0.7	1	$-0.36$	0	0.5	5
3*b*								10
3*c*								25
4*a*	$4\cdot t$	$4\cdot t$	0.5	0.7	$-0.69$	$-0.36$	1	5
4*b*								10
4*c*								25
5*a*	$4\cdot t$	$4\cdot t$	0.7	1	$-0.36$	0	1	5
5*b*								10
5*c*								25
6*a*	$t^{-0.5}$	$4\cdot t$	0.5	0.7	$-0.69$	$-0.36$	0.5	5
6*b*								10
6*c*								25
7*a*	$t^{-0.5}$	$4\cdot t$	0.7	1	$-0.36$	0	0.5	5
7*b*								10
7*c*								25
8*a*	$t^{-0.5}$	$4\cdot t$	0.5	0.7	$-0.69$	$-0.36$	1	5
8*b*								10
8*c*								25
9*a*	$t^{-0.5}$	$4\cdot t$	0.7	1	$-0.36$	0	1	5
9*b*								10
9*c*								25
10*a*	$4\cdot t$	$t^{-0.5}$	0.5	0.7	$-0.69$	$-0.36$	0.5	5
10*b*								10
10*c*								25
11*a*	$4\cdot t$	$t^{-0.5}$	0.7	1	$-0.36$	0	0.5	5
11*b*								10
11*c*								25
12*a*	$4\cdot t$	$t^{-0.5}$	0.5	0.7	$-0.69$	$-0.36$	1	5
12*b*								10
12*c*								25
13*a*	$4\cdot t$	$t^{-0.5}$	0.7	1	$-0.36$	0	1	5
13*b*								10
13*c*								25

Scen. =Scenario; log=natural logarithm; $\lambda _{0,PE}(t)$, $\lambda _{0,CE}(t)$, $HR_{PE}$, and $HR_{CE}$ are the baseline hazards and hazard ratios for the primary endpoint (*PE*) and competing event (*CE*), respectively

In Table 1 the simulation scenarios are listed. In Column 2 the baseline hazard for the primary event of interest is given and in Column 3 the baseline hazard for the competing event. Columns 4 and 5 show the assumed hazard ratio for the primary event and the competing event, respectively. Columns 6 and 7 show the assumed logarithmic hazard ratio for the primary event and the competing event, respectively. In Column 8 the assumed variance for the gamma distributed frailty is given ($\theta$). In the last column the cluster count is given.

For Scenario 1 the baseline hazards for the two event types are the same. The hazard ratios differ but point into the same direction. Here, also no center-specific distribution is assumed, i.e. $\theta =0$. Nevertheless, three different cluster counts are considered for all scenarios which are depicted in scenarios a-c. Within Scenario 2 a moderate center-specific effect is assumed. In Scenario 3 the hazard ratios for the two event types change, i.e. an effect is only assumed for the primary event but not for the competing event.

Within Scenarios 4 and 5 the difference between centers increases since a higher frailty variance is assumed. For Scenarios 6-13 the baseline hazards change for either the primary event of interest or the competing event but else similar hazard ratios and frailty variances are assumed.

For all scenarios 250 individuals in total per data set were generated with about 125 in each treatment group, i.e. allocated using a binomial distribution. A follow-up of two years was assumed. For each scenario 2000 data sets were simulated and analyzed.

For performance comparison we considered the mean and standard deviation of estimated logarithmized hazard ratios for the primary event of interest as well as the corresponding absolute bias, square root of the mean squared error, and the empirical power.

Our primary interest is hence how the underlying treatment effect is estimated by the different models. Although it might also be of interest to evaluate the model performances regarding the cluster effect estimation, this cannot be done adequately. The proposed methods do not estimate the number of clusters but the cluster variance. The methods differ in their estimation approach for the cluster variance and are also different from our data simulation approach and thus it cannot be defined what a misspecification for the cluster structure would mean.

### Application data set

To further illustrate the four approaches we used a data set (*center*) that is publicly available within the package named *crrSC* [23] of the statistic software R. The data set named *center* consists of multi-center bone marrow transplantation data and includes 400 patients from 153 transplant centers. The primary event of interest is “Acute or chronic Graft-versus-Host-Disease (GvHD)”. The competing event is “death” (without GvHD). We are interested in the influence of the source of stem cells, i.e. peripheral blood vs. bone marrow.

## Results

### Results of simulation study

**Fig. 1:**
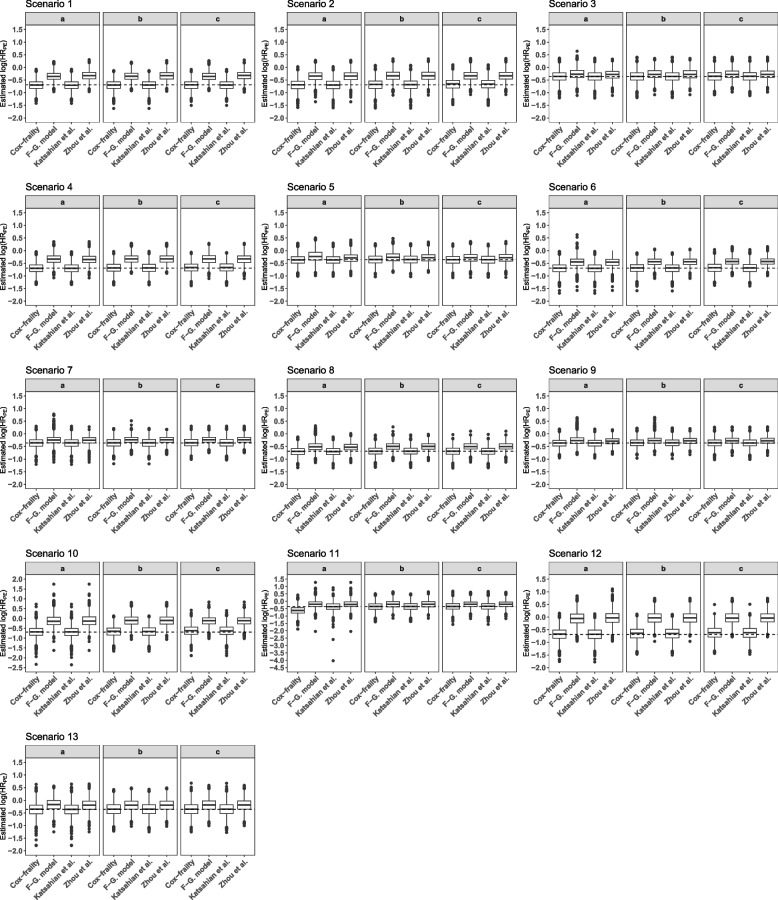
Results of the simulation study: Boxplots for the estimated effects for the primary outcome. Dashed line = true underlying simulated effect; $log(HR_{PE})$ = logarithmic hazard ratio for primary endpoint

In Tables 2 and 3 the results of the simulation study are displayed. Figure 1 further illustrates the results.

**Table 2:** Simulation Results

Scen.	mean amount of events (sd)		Estimated log($HR_{PE}$) (sd) for primary endpoint				Power			
	primary	competing	Cox-frailty	F-G. model	Katsahian et al.	Zhou et al.	Cox-frailty	F-G. model	Katsahian et al.	Zhou et al.
1*a*	114.73 (7.86)	135.26 (7.86)	-0.70 (0.20)	-0.36 (0.18)	-0.70 (0.20)	-0.33 (0.19)	0.95	0.52	0.95	0.53
1*b*	114.92 (7.83)	135.07 (7.82)	-0.70 (0.20)	-0.35 (0.18)	-0.71 (0.20)	-0.33 (0.19)	0.95	0.51	0.96	0.48
1*c*	114.68 (7.98)	135.31 (7.98)	-0.70 (0.20)	-0.36 (0.17)	-0.70 (0.20)	-0.32 (0.19)	0.95	0.53	0.95	0.42
2*a*	92.49 (17.62)	108.47 (21.17)	-0.70 (0.22)	-0.35 (0.21)	-0.70 (0.23)	-0.35 (0.21)	0.89	0.35	0.89	0.52
2*b*	92.50 (13.41)	108.69 (14.76)	-0.68 (0.22)	-0.33 (0.21)	-0.68 (0.22)	-0.33 (0.21)	0.88	0.34	0.88	0.42
2*c*	92.14 (10.35)	108.29 (11.11)	-0.66 (0.22)	-0.33 (0.20)	-0.66 (0.22)	-0.33 (0.20)	0.85	0.34	0.85	0.41
3*a*	93.85 (17.25)	111.51 (20.84)	-0.36 (0.22)	-0.26 (0.22)	-0.37 (0.22)	-0.29 (0.20)	0.39	0.24	0.40	0.43
3*b*	93.73 (13.24)	111.62 (14.50	-0.36 (0.22)	-0.27 (0.21)	-0.36 (0.22)	-0.28 (0.21)	0.38	0.25	0.39	0.35
3*c*	93.48 (20.23)	111.29 (10.99)	-0.35 (0.22)	-0.27 (0.20)	-0.35 (0.22)	-0.27 (0.20)	0.36	0.24	0.37	0.30
4*a*	106.29 (12.17)	124.77 (13.76)	-0.70 (0.20)	-0.34 (0.20)	-0.70 (0.20)	-0.35 (0.19)	0.94	0.40	0.94	0.56
4*b*	106.00 (10.09)	124.72 (11.11)	-0.68 (0.20)	-0.33 (0.19)	-0.68 (0.20)	-0.33 (0.19)	0.92	0.39	0.92	0.47
4*c*	106.15 (8.58)	124.75 (8.98)	-0.67 (0.20)	-0.34 (0.19)	-0.67 (0.20)	-0.34 (0.20)	0.91	0.40	0.91	0.44
5*a*	107.07 (11.70)	127.25 (13.17)	-0.37 (0.19)	-0.22 (0.23)	-0.37 (0.19)	-0.30 (0.19)	0.46	0.25	0.46	0.47
5*b*	106.75 (9.87)	127.21 (10.77)	-0.36 (0.20)	-0.26 (0.21)	-0.36 (0.20)	-0.28 (0.19)	0.42	0.27	0.42	0.38
5*c*	106.91 (8.41)	127.22 (8.98)	-0.36 (0.20)	-0.29 (0.19)	-0.36 (0.20)	-0.29 (0.19)	0.42	0.30	0.42	0.34
6*a*	114.02 (34.76)	77.86 (21.72)	-0.70 (0.21)	-0.46 (0.20)	-0.70 (0.21)	-0.47 (0.19)	0.92	0.64	0.92	0.84
6*b*	114.98 (24.93)	77.41 (15.57)	-0.69 (0.20)	-0.45 (0.17)	-0.70 (0.20)	-0.45 (0.17)	0.94	0.64	0.94	0.83
6*c*	113.78 (16.21)	77.79 (11.03)	-0.68 (0.20)	-0.43 (0.16)	-0.69 (0.20)	-0.43 (0.16)	0.93	0.65	0.93	0.82
7*a*	121.03 (35.88)	75.95 (22.49)	-0.36 (0.20)	-0.24 (0.18)	-0.36 (0.20)	-0.26 (0.16)	0.48	0.22	0.48	0.51
7*b*	122.04 (25.71)	75.20 (16.13)	-0.36 (0.19)	-0.24 (0.15)	-0.36 (0.19)	-0.24 (0.15)	0.47	0.22	0.48	0.44
7*c*	120.75 (16.64)	75.77 (11.21)	-0.35 (0.19)	-0.24 (0.15)	-0.36 (0.19)	-0.24(0.15)	0.46	0.19	0.47	0.40
8*a*	134.29 (28.71)	89.77 (18.07)	-0.70 (0.18)	-0.50 (0.19)	-0.70 (0.18)	-0.53 (0.16)	0.97	0.80	0.97	0.93
8*b*	134.28 (20.30)	89.43 (13.93)	-0.69 (0.18)	-0.50 (0.16)	-0.69 (0.18)	-0.50 (0.16)	0.97	0.84	0.97	0.91
8*c*	134.09 (13.91)	89.70 (10.36)	-0.69 (0.18)	-0.50 (0.15)	-0.69 (0.18)	-0.50 (0.15)	0.97	0.84	0.97	0.92
9*a*	142.90 (29.48)	85.39 (19.38)	-0.37 (0.17)	-0.25 (0.20)	-0.37 (0.17)	-0.29 (0.15)	0.57	0.33	0.57	0.62
9*b*	142.83 (20.77)	85.08 (14.60)	-0.35 (0.17)	-0.27 (0.16)	-0.35 (0.17)	-0.28 (0.15)	0.55	0.33	0.55	0.54
9*c*	142.68 (14.16)	85.32 (10.74)	-0.36 (0.17)	-0.28 (0.15)	-0.36 (0.18)	-0.28 (0.15)	0.64	0.36	0.54	0.51
10*a*	64.25 (19.46)	125.17 (37.09)	-0.68 (0.29)	-0.12 (0.30)	-0.69 (0.29)	-0.11 (0.30)	0.72	0.11	0.73	0.21
10*b*	63.83 (14.39)	126.16 (26.09)	-0.66 (0.28)	-0.11 (0.27)	-0.67 (0.28)	-0.10 (0.27)	0.69	0.07	0.69	0.14
10*c*	64.09 (10.31)	124.80 (16.82)	-0.63 (0.28)	-0.12 (0.24)	-0.63 (0.28)	-0.12 (0.24)	0.62	0.06	0.63	0.09
11*a*	61.84 (19.85)	132.82 (37.09)	-0.36 (0.29)	-0.20 (0.27)	-0.36 (0.30)	-0.21 (0.27)	0.27	0.12	0.27	0.29
11*b*	61.17 (14.71)	133.86 (26.43)	-0.36 (0.28)	-0.21 (0.25)	-0.36 (0.28)	-0.21 (0.25)	0.26	0.11	0.26	0.21
11*c*	61.66 (10.29)	132.56 (17.13)	-0.35 (0.28)	-0.21 (0.24)	-0.35 (0.28)	-0.21 (0.24)	0.24	0.10	0.23	0.14
12*a*	73.67 (17.19)	148.09 (30.11)	-0.68 (0.26)	-0.06 (0.26)	-0.68 (0.26)	-0.03(0.27)	0.77	0.10	0.78	0.15
12*b*	73.29 (12.86)	147.87 (21.28)	-0.66 (0.25)	-0.03 (0.25)	-0.66 (0.25)	-0.03 (0.25)	0.74	0.06	0.75	0.11
12*c*	73.70 (9.89)	147.71 (14.61)	-0.62 (0.26)	-0.05 (0.24)	-0.62 (0.26)	-0.04 (0.24)	0.68	0.05	0.69	0.07
13*a*	68.95 (18.09)	157.49 (30.12)	-0.36 (0.27)	-0.18 (0.24)	-0.36 (0.27)	-0.19 (0.24)	0.29	0.10	0.29	0.27
13*b*	68.53 (13.31)	157.29 (21.26)	-0.36 (0.25)	-0.19 (0.23)	-0.36 (0.26)	-0.19 (0.24)	0.28	0.11	0.28	0.19
13*c*	68.97 (9.98)	157.11 (14.48)	-0.35 (0.26)	-019 (0.24)	-0.35 (0.26)	-0.19 (0.24)	0.26	0.12	0.26	0.16

Scen. =Scenario; log=natural logarithm; HR=hazard ratio; sd=standard deviation; F-G.=Fine-Gray

**Table 3:** Simulation Results

Scen.	bias for primary log($HR_{PE}$)				$\sqrt{\text {mean squared error}}$ for primary log($HR_{PE}$)			
	Cox-frailty	F-G. model	Katsahian et al.	Zhou et al.	Cox-frailty	F-G. model	Katsahian et al.	Zhou et al.
1*a*	-0.01	0.34	-0.01	0.37	0.20	0.38	0.20	0.41
1*b*	-0.01	0.34	-0.01	0.36	0.20	0.39	0.20	0.41
1*c*	-0.01	0.33	-0.01	0.37	0.20	0.38	0.20	0.42
2*a*	-0.01	0.34	-0.01	0.34	0.22	0.40	0.23	0.40
2*b*	0.01	0.36	0.01	0.36	0.22	0.42	0.22	0.42
2*c*	0.03	0.36	0.03	0.36	0.22	0.41	0.23	0.41
3*a*	-0.01	0.09	-0.01	0.07	0.22	0.24	0.22	0.21
3*b*	-0.00	0.08	-0.00	0.08	0.22	0.22	0.22	0.22
3*c*	0.00	0.08	0.00	0.08	0.22	0.21	0.22	0.21
4*a*	-0.01	0.35	-0.01	0.34	0.20	0.41	0.20	0.40
4*b*	0.01	0.36	0.01	0.36	0.20	0.41	0.20	0.41
4*c*	0.02	0.35	-0.01	0.35	0.21	0.40	0.21	0.40
5*a*	-0.01	0.14	-0.01	0.06	0.19	0.27	0.19	0.20
5*b*	0.00	0.10	0.00	0.07	0.20	0.23	0.20	0.20
5*c*	-0.01	0.07	-0.01	0.07	0.20	0.21	0.20	0.20
6*a*	-0.01	0.23	-0.01	0.22	0.21	0.30	0.21	0.29
6*b*	0.00	0.25	-0.00	0.25	0.20	0.30	0.20	0.30
6*c*	0.01	0.26	0.00	0.26	0.20	0.30	0.20	0.30
7*a*	-0.01	0.11	-0.01	0.10	0.20	0.22	0.20	0.19
7*b*	-0.00	0.11	-0.00	0.11	0.29	0.19	0.19	0.19
7*c*	0.01	0.12	0.00	0.12	0.19	0.30	0.19	0.19
8*a*	-0.01	0.19	-0.01	0.17	0.18	0.27	0.18	0.23
8*b*	0.01	0.19	0.00	0.19	0.18	0.25	0.18	0.25
8*c*	0.00	0.19	-0.00	0.19	0.18	0.25	0.18	0.25
9*a*	-0.01	0.11	-0.01	0.06	0.17	0.22	0.17	0.16
9*b*	0.00	0.09	0.00	0.08	0.17	0.19	0.17	0.17
9*c*	-0.00	0.08	-0.01	0.08	0.17	0.17	0.17	0.17
10*a*	0.01	0.57	0.01	0.58	0.29	0.64	0.29	0.66
10*b*	0.03	0.59	0.02	0.59	0.28	0.65	0.28	0.65
10*c*	0.07	0.58	0.06	0.58	0.29	0.62	0.29	0.62
11*a*	-0.00	0.15	-0.00	0.15	0.29	0.31	0.30	0.30
11*b*	-0.00	0.15	-0.00	0.15	0.28	0.30	0.28	0.30
11*c*	0.01	0.15	0.01	0.15	0.28	0.28	0.28	0.28
12*a*	0.02	0.64	0.01	0.66	0.26	0.69	0.26	0.71
12*b*	0.04	0.66	0.03	0.66	0.25	0.70	0.26	0.70
12*c*	0.07	0.66	0.07	0.66	0.27	0.70	0.27	0.70
13*a*	-0.00	0.18	-0.00	0.16	0.27	0.30	0.27	0.29
13*b*	0.00	0.17	0.00	0.16	0.25	0.29	0.26	0.29
13*c*	0.00	0.17	0.00	0.17	0.26	0.29	0.26	0.29

Scen. =Scenario; log=natural logarithm; F-G= Fine-Gray; HR=Hazard ratio

Note that the models by Fine and Gray, Katsahian et al., and Zhou et al. can only estimate the least false parameter due to our simulation set-up (which is basically based on the Cox model with frailty). This is due to the property that these models originally aim to get a prognosis taking into acount the competing event and do not focus on the estimation of the treatment effect. However, we want to evaluate whether one of the models can also produce an unbiased treatment effect estimate.

It can be seen that independent of the cluster structures, i.e. cluster count or correlation, the Cox model with frailty and the method by Katsahian et al. yield the smallest bias and square root of the mean squared error. Overall, these two models show very similar estimation results. The results of the Fine and Gray model and the model by Zhou et al. are also quite similar. Hence, bias and square root of the mean squared error coincide but are in some scenarios considerably higher compared to the results within the Cox model or the model by Katsahian et al. The bias and square root of the mean squared error within the Fine and Gray model and the model by Zhou et al. is higher in scenarios where the assumed hazard ratio for the competing event is not equal to 1.

The results seen for the bias or square root of the mean squared error are also reflected in the empirical power. The power for the Cox model and the model by Katsahian et al. coincide and exceed those of the Fine and Gray model and the model by Zhou et al. in nearly all scenarios (depending on the underlying simulated hazard ratio). The power of the model by Zhou et al. is higher than that observed for the model by Fine and Gray in all scenarios except the first where no cluster variance is assumed.

For the Cox model with frailty, 1 and 2 data sets produced an convergence error in Scenarios 10a and 11a, respectively and hence could not be included in the comparison.

### Results of application

The results of the four different models for the application data set are given in Table 4. The source of stem cells was peripheral blood for 178 (44.5$\%$) patients and bone marrow for 222 (55.5$\%$) patients. For patients with peripheral blood as source of stem cells 79 (44.4$\%$) primary events of interest were observed and 36 (20.2$\%$) competing death events. For patients with bone marrow as source of stem cells 115 (51.8$\%$) primary events of interest were observed and 38 (17.1$\%$) competing death events. Median follow-up was estimated using the reverse Kaplan-Meier method and is 1639 days, the maximum observed event time for the primary event of interest is 2668 days, and maximum observed (censoring) time is 5138 days. The estimated hazard ratio for the primary event of interest is 0.85 for the Cox model with frailty but for the other approaches it is 0.82. For the approach by Fine and Gray and Zhou et al. this is in line with the results of our simulation study and hence the method by Zhou et al. seems to be an adequate extension for the Fine and Gray model when a cluster structure is present. Interestingly, the results gained by the approach by Katsahian et al. deviates more from the Cox-frailty results than seen in the simulation study. The main difference between our simulation study and the example is the number of clusters. Hence, this might be one reason but it could also be the cluster structure itself, i.e. cluster correlation and underlying distribution. To explore this, we altered the number of clusters in the example data by joining different clusters and present the results of these additional analyses in the appendix. However, the number of clusters seems not to be the reason for the difference between the Cox model results and the results by the approach by Katsahian et al. since the pattern of effect estimates remains the same. Another difference might be that the cause-specific hazards are not proportional but the subdistribution hazards.

**Table 4:** Application Results

Model	$HR_{PE}$ (95 $\%$-CI)	*p*-value
Cox-frailty	0.85 (0.63, 1.16)	0.31
F-G model	0.82 (0.63, 1.07)	0.15
Katsahian et al.	0.82 (0.60, 1.12)	0.22
Zhou et al.	0.82 (0.63, 1.08)	0.16

F-G= Fine-Gray; HR=Hazard ratio; PE=Primary endpoint; CI=confidence interval

## Discussion

The analysis of a time to event endpoint where competing events and a cluster structure are present is a challenging task in cardiovascular or oncologic trials. Therefore, we compared four different methods that were proposed for those studies to give an overview of their properties in different clinical data situations. Here, the focus was on treatment effect estimation and not on prognosis which was already the focus of the main publications of the newly proposed methods [4, 6]. Hence, we extended the systematic comparison of the proposed methods to the effect evaluation to gain more insights in whether the proposed methods can also be used for unbiased treatment effect estimation and not only prognosis. The proposed methods differ in their performance and assumptions.

The cause-specific Cox-frailty model estimates the effect of the treatment on the rate of occurrence of the primary outcome of interest in those subjects who are currently free of any events (and conditional on the frailty). Since the simulation study does not include correlated event types, the true underlying effect for the primary endpoint can be estimated without bias, as seen in the results of the simulation study. Hence, the cause-specific Cox model is a good choice and can be recommended if the treatment effect on the rate of occurrence is of interest rather than prediction as it was already mentioned by Austin et al. and Allison [2, 24].

However, the performance of the model should be evaluated in scenarios where the endpoints are more correlated.

The Fine and Gray model allows one to estimate the effect of the treatment on the absolute risk of the primary outcome of interest over time, i.e. if one is more interested in prognosis. As described by Allison [24] the model may not be a good choice if one is interested in an unbiased treatment effect in randomized clinical trials for the primary event of interest. This is again supported by our simulation study. Moreover, it does not incorporate the cluster structure and hence we recommend to use the following model.

Katsahian et al. described a proportional subdistribution hazards model which is a frailty model. The model is also described as an extension of the Fine and Gray model to allow cluster structure, but produces unbiased treatment effects for the primary endpoint of interest for randomized clinical trials as also seen in the results of our simulation study. The model allows to estimate the cluster effect, as well as to incorporate this effect in prognostic analyses [4]. The latter is an advantage over the Cox-frailty model. Thus, this model can be recommended to estimate the treatment effect in clinical trials with competing events and a given cluster structure. However, it might be of interest to evaluate its performance in a setting where the event types are (more) correlated.

The model by Zhou et al. shows similar results in our simulation study as for the Fine and Gray model. Since this model was also described as extension of the latter, this is not surprising. We therefore conclude that if one is interested in the prognosis for the patients rather than an unbiased treatment effect on occurrence rates to use this model if a cluster structure is present in the data with competing risks.

Zhou et al. did conclude the same for their analysis. I.e. their simulation showed the potential bias and loss of power in hypothesis testing [6]. They hypothesized that this may arise from ignoring within-cluster correlations in variance estimation [6]. They also referred to the model by Katsahian et al. as an alternative. They further described that their method is particularly useful in applications with small groups of correlated observations where the correlation is mainly a confounding factor.

The application supported the results that the model by Zhou et al. is an appropriate extension of the Fine and Gray model in the case of a multi-center trial. However, it also supports that more complex simulation studies and evaluation of application might be necessary to shed light on the model by Katsahian et al.

Moreover, other approaches of interest in future work might be additive models or models using copulas [13, 14].

## Conclusion

In conclusion, for clinical studies where two groups shall be compared regarding a time to event endpoint where competing events and a cluster structure are present the approach by Katsahian et al. can be recommended since it allows unbiased effect estimation and prognosis [4]. However, extended simulation studies are necessary to confirm its application in a broader range of data settings. For unbiased treatment effect estimation without the focus on prognosis, the Cox model with frailty can be used.

## Supplementary Information

**Additional file 1.** R code for the simulation study and example data set as well as further results for the example data set.

## Data Availability

Simulated data can be obtained from the authors (a.ozga@uke.de) upon request. R code for the simulation study can be found in the Additional file. The example data set (center) is freely available within R in the package crrSC https://rdrr.io/cran/crrSC/man/center.html (https://cran.r-project.org/web/packages/crrSC/crrSC.pdf). The example data set can also be found in the appendix.

## Abbreviations

C: Control
CE: Competing event
CI: Confidence interval
F.-G.: Fine and Gray
GvHD: Graft versus host disease
HR: Hazard ratio
I: Intervention
log: Logarithm
PE: Primary endpoint
Scen.: Scenario
sd: Standard deviation
