# Supplementary material for: Systematic comparison of approaches to analyze clustered competing risks data
Source: BMC Med Res Methodol. 2023 Apr 10;23:86. doi: 10.1186/s12874-023-01908-6 (PMC10084627; doi:10.1186/s12874-023-01908-6)
Supplement: Supplementary file 1 — Additional file 1. R code for the simulation study and example data set as well as further results for the example data set. [file 12874_2023_1908_MOESM1_ESM.zip › 12874_2023_1908_MOESM1_ESM/AdditionalFile.pdf]

RESEARCH

Additional file for: 'Systematic comparison of approaches to analyze clustered competing risks data'

Sabrina Schnitt, Anika Buchholz and Ann-Kathrin Ozga

Full list of author information is available at the end of the article

1 Addtional results for application

The tables 1,2, and 3 show the results of the effect estimation if some of the given clusters in the application data are joined together such that only 5, 20, or 25 clusters are considered, respectively.

Table 1 Application results with nr. of clusters = 5

Model	HR_{PE} (95 %-CI)	p-value
Cox-frailty	0.88 (0.66, 1.17)	0.37
F-G model	0.82 (0.63, 1.07)	0.15
Katsahian et al.	0.82 (0.62, 1.10)	0.18
Zhou et al.	0.82 (0.51, 1.32)	0.42

F-G= Fine-Gray; HR=Hazard ratio; PE=Primary endpoint; CI=confidence interval

Table 2 Application results with nr. of clusters = 10

Model	HR_{PE} (95 %-CI)	p-value
Cox-frailty	0.88 (0.66, 1.17)	0.37
F-G model	0.82 (0.63, 1.07)	0.15
Katsahian et al.	0.82 (0.62, 1.10)	0.18
Zhou et al.	0.82 (0.56, 1.23)	0.34

F-G= Fine-Gray; HR=Hazard ratio; PE=Primary endpoint; CI=confidence interval

Table 3 Application results with nr. of clusters = 25

Model	HR_{PE} (95 %-CI)	p-value
Cox-frailty	0.88 (0.66, 1.17)	0.37
F-G model	0.82 (0.63, 1.07)	0.15
Katsahian et al.	0.82 (0.62, 1.10)	0.18
Zhou et al.	0.82 (0.58, 1.18)	0.29

F-G= Fine-Gray; HR=Hazard ratio; PE=Primary endpoint; CI=confidence interval
